# Development of a Quantitative PCR Assay for *Arcobacter* spp. and its Application to Environmental Water Samples

**DOI:** 10.1264/jsme2.ME18052

**Published:** 2018-09-29

**Authors:** Rajani Ghaju Shrestha, Yasuhiro Tanaka, Bikash Malla, Sarmila Tandukar, Dinesh Bhandari, Daisuke Inoue, Kazunari Sei, Jeevan B. Sherchand, Eiji Haramoto

**Affiliations:** 1 Department of Natural, Biotic and Social Environment Engineering, University of Yamanashi 4–3–11 Takeda, Kofu, Yamanashi 400–8511 Japan; 2 Department of Environmental Sciences, University of Yamanashi 4–4–37 Takeda, Kofu, Yamanashi 400–8510 Japan; 3 Institute of Medicine, Tribhuvan University Maharajgunj, Kathmandu Nepal; 4 Division of Sustainable Energy and Environmental Engineering, Osaka University 2–1 Yamadaoka, Suita, Osaka 565–0871 Japan; 5 Department of Health Science, Kitasato University 1–15–1 Kitasato, Minami-ku, Sagamihara, Kanagawa 252–0373 Japan; 6 Interdisciplinary Center for River Basin Environment, University of Yamanashi 4–3–11 Takeda, Kofu, Yamanashi 400–8511 Japan

**Keywords:** *Arcobacter* spp., Kathmandu Valley, next-generation sequencing, quantitative PCR, water source

## Abstract

*Arcobacter* spp. are emerging pathogens associated with gastroenteritis in humans. The objective of this study was to develop a highly sensitive and broadly reactive quantitative PCR (qPCR) assay for *Arcobacter* spp. and to apply the developed assay to different water sources in the Kathmandu Valley, Nepal. Fifteen samples to be analyzed by next-generation sequencing were collected from 13 shallow dug wells, a deep tube well, and a river in the Kathmandu Valley in August 2015. Among the 86 potential pathogenic bacterial genera identified, *Acinetobacter*, *Pseudomonas*, *Flavobacterium*, and *Arcobacter* were detected with relatively high abundance in 15, 14, 12, and 8 samples, respectively. A primer pair was designed with maximal nucleotide homologies among *Arcobacter* spp. by comparing the sequences of 16S rRNA genes. These primers were highly specific to most of the known species of *Arcobacter* and quantified between 1.0×10^1^ and 6.4×10^6^ copies reaction^−1^ and sometimes detected as few as 3 copies reaction^−1^. The qPCR assay was used to quantify *Arcobacter* spp. in bacterial DNA in not only the above 15 water samples, but also in 33 other samples collected from 15 shallow dug wells, 6 shallow tube wells, 5 stone spouts, 4 deep tube wells, and 3 springs. Thirteen (27%) out of 48 samples tested were positive for *Arcobacter* spp., with concentrations of 5.3–9.1 log copies 100 mL^−1^. This qPCR assay represents a powerful new tool to assess the prevalence of *Arcobacter* spp. in environmental water samples.

In developing countries such as Nepal, particularly in the Kathmandu Valley, water supply cannot be entirely fulfilled due to the inability of the system to match the demands of a growing population. In the Kathmandu Valley, 34% of individuals use alternative water sources, such as ground, jar, tanker, rain, spring, stone spout, and river water as domestic supplies (41). Various microbiological studies on water samples have been conducted (17, 20, 21, 26, 29, 35, 38, 42, 45), resulting in the detection of fecal indicator bacteria, pathogenic bacteria, protozoa, and viruses in ground, surface, irrigation, and even jar water (which is perceived as potable). In our previous study conducted in the Kathmandu Valley, a next-generation sequencing (NGS) analysis demonstrated that *Acinetobacter*, *Arcobacter*, and *Clostridium* were highly abundant in ground and river water samples, and that *Arcobacter* was the second most dominant pathogenic bacterium and present in all 16 samples tested, with a maximum abundance ratio of 17.43% (17).

Bacteria in the genus *Arcobacter* are Gram-negative rods and are classified into the family *Campylobacteraceae* (46). A bacterium within the genus was initially isolated in 1977 from bovine and pig fetuses (13), and 22 species have since been recognized (15, 34, 50). Some members of the genus, particularly *Arcobacter butzleri*, are predominantly associated with gastroenteritis, septicemia, mastitis, reproductive disorders, and abortion in livestock, and are now emerging as potential foodborne and waterborne pathogens (9, 12).

Most *Arcobacter* spp. are reported as being commensal in the gastrointestinal tracts of animals, such as cattle, pigs, chickens, sheep, and horses (23), and the feces of these animals may be a source of water contamination. The occurrence of *Arcobacter* spp. in various foods, such as chicken, pork, beef, unpasteurized milk, shellfish, and restaurant meals, suggests the fecal-oral transmission of these bacteria (9). The distribution patterns of *Arcobacter* spp. have been reported in different countries, including Turkey, Spain, South Africa, USA, Germany, and Italy, and bacteria have been recovered from various types of water, including waste, sea, surface, ground, and recreational water (7, 9, 25). Therefore, the presence of *Arcobacter* in water and food is a potential health risk for humans and animals.

*A. butzleri*, *A. cryaerophilus*, *A. trophiarum*, and *A. thereius* are considered to be representative pathogenic species in the genus *Arcobacter* (9, 11, 24). Furthermore, a study that focused on meat contamination identified six other pathogenic *Arcobacter* spp.: *A. skirrowii*, *A. cibarius*, *A. defluvii*, *A. ellisii*, *A. mytili*, and *A. molluscorum* (19). The complete genome sequencing of *A. butzleri* strain RM4018 (GenBank accession number, CP000361) revealed the presence of nine virulence determinants: *cadF*, *ciaB*, *cj1349*, *mviN*, *pldA*, *tlyA*, *hecA*, *hecB*, and *irgA* (36). These genes are associated with pathogenicity because they function in processes including adhesion, invasion, and cytotoxicity, and 14 *Arcobacter* species were identified as possessing these pathogenic characteristics. These species include the above described *Arcobacter* spp. along with *A. nitrofigilis*, *A. venerupis*, *A. cloacae*, and *A. suis* (15).

Many PCR assays have been developed for the detection of *Arcobacter* spp. (1, 5, 6, 11, 18, 19, 22, 24, 49); however, none have the ability to detect all the *Arcobacter* species that possess pathogenic characteristics. These assays have targeted some pathogenic species of *Arcobacter* associated with food (18, 19) as well as humans and animals (5, 6, 12, 22, 24, 49), along with other species (1, 5). The high abundance of *Arcobacter* spp. in water samples (17), and their prevalence in food, marks them as emerging pathogens, and limitations in the detection of some species justifies the development of a PCR assay with the ability to detect the presence of all members of the genus.

Therefore, we aimed to develop a highly sensitive and broadly reactive quantitative PCR (qPCR) assay for *Arcobacter* spp. and apply the developed assay to the quantification of *Arcobacter* spp. in different water samples from the Kathmandu Valley.

## Materials and Methods

### Collection of water samples and bacterial DNA extraction

In August 2015, 15 samples were collected from 13 shallow dug wells, a deep tube well, and a river in the Kathmandu Valley. Samples were collected in sterile 100-mL plastic bottles, stored in bags containing ice packs, and transported to the laboratory as quickly as possible.

After sample filtration through a 0.2-μm membrane filter (NALGENE, Tokyo, Japan), the filter was placed in a 50-mL plastic tube and 5 mL of Tris-EDTA buffer was added. Following shaking and vortex mixing, 70 μL of the sample was used for DNA extraction using a CicaGeneus DNA extraction kit (Kanto Chemical, Tokyo, Japan), as described previously (17).

### Bacterial community characterization by the NGS analysis

Samples were analyzed by 16S rRNA gene sequencing using the universal primers 515F and 806R (4, 44). Amplicons were sent to FASMAC (Atsugi, Japan) for sequencing using a MiSeq gene sequencer (Illumina, San Diego, CA, USA). Chimera checks and quality control were performed by FASMAC. The operational taxonomic units obtained were analyzed based on the bacterium domain, phylum, family, and genus. When at least one species of any genus was categorized as bio-safety level 2 or 3 by the American Biological Safety Association (https://my.absa.org/tiki-index.php?page=Riskgroups), the entire genus was considered to be potentially pathogenic.

### Primer design for *Arcobacter* spp.

**Fig. 1:**
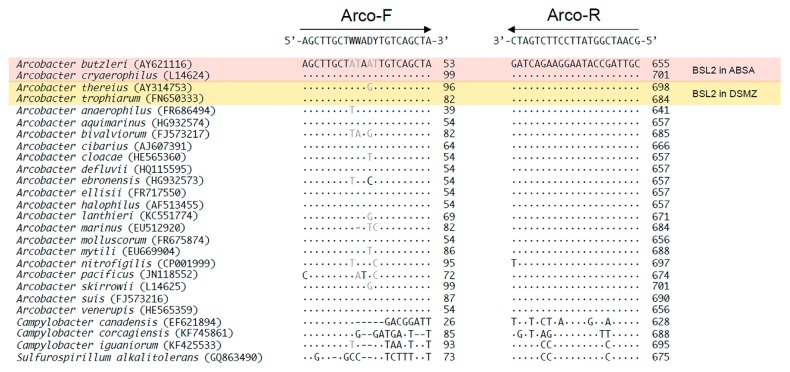
Alignment of 26 nucleotide sequences of 16S rRNA genes from the family *Campylobacteraceae*. GenBank accession numbers follow species names. ABSA, American Biological Safety Association; DSMZ, Deutsche Sammlung von Mikroorganismen und Zellkulturen GmbH.

Twenty-six sequences of 16S rRNA genes from the family *Campylobacteraceae* were obtained from the GenBank database and aligned using multiple alignment in GENETYX 9.0 software (GENETYX Corporation, Tokyo, Japan). Twenty-two out of the 26 sequences belonged to different species of *Arcobacter*, whereas the remaining 4 were of non-*Arcobacter* species from the same family as *Arcobacter*. As shown in Fig. 1, comparisons of the aligned sequences allowed the localization of regions displaying the maximum nucleotide homology among *Arcobacter* spp. and, in turn, sufficient nucleotide differences from heterologous species. The forward primer Arco-F (5′-AGCTTGCTWWADYTGTCAGCTA-3′, corresponding to nucleotide positions 32–53 of the *A. butzleri* 16S rRNA gene) and reverse primer Arco-R (5′-GCAATCGGTATTCCTTCT GATC-3′, corresponding to nucleotide positions 634–655 of the *A. butzleri* 16S rRNA gene) were selected for the potential selective amplification of 624–625-bp DNA fragments of the 16S rRNA gene of the genus *Arcobacter*. The designed primers were also aligned with the following non-*Arcobacter* sequences: *Helicobacter*, *Salmonella*, *Escherichia*, *Yersinia*, *Streptococcus*, *Enterococcus*, and *Wolinella*.

### Specificity of designed primers by the sequencing analysis

Bacterial DNA extracted from water samples was used to assess the specificity of the designed primers. Conventional PCR was performed using a 50-μL reaction mixture containing 2 μL of bacterial DNA, 0.25 μL each of 50 pmol μL^−1^ of Arco-F and Arco-R primers, 25 μL of a SapphireAmp Fast PCR Master Mix (Takara Bio, Otsu, Japan), and 22.5 μL of ultrapure water. The thermal conditions used were as follows: 94°C for 1 min, followed by 35 cycles at 98°C for 5 s, at 55°C for 5 s, and at 72°C for 10 s, and finally at 72°C for 10 s. Agarose gel electrophoresis was performed to confirm the amplification of PCR products of the expected length (approx. 600 bp). Amplified fragments were purified using a NucleoSpin Gel and PCR Clean-up Kit (Macherey-Nagel, Bethlehem, PA, USA) and ligated into a pMD19 T-vector (Takara Bio). The resulting recombinant plasmids were transformed into competent *E. coli* DH5α cells (Takara Bio).

PCR amplification was then performed in a 50-μL volume from a single colony of *E. coli*, using 0.25 μL each of 50 pmol μL^−1^ pMD-F (5′-CACGCCTGCCGTTCGACGAT-3′) and pMD-R (5′-CGCGCGGATCTTCCAGAGAT-3′) primers, which are specific to the vector sites flanking the inserted DNA, 29.5 μL of a SapphireAmp Fast PCR Master Mix, and 20 μL of ultrapure water and using the same thermal conditions as those described above. Agarose gel electrophoresis was performed using 5 μL of amplified PCR products, and DNA purification was conducted with a NucleoSpin Gel and PCR Clean-up Kit using 45 μL of amplified PCR products. The purified DNA products exhibiting positive bands on the agarose gel were selected and sequenced using the pMD-F primer as described previously (45). The data obtained by the sequencing of samples were compared with those in the GenBank database using the BLAST search program (http://www.ncbi.nlm.nih.gov/blast/).

### Sensitivity of the qPCR assay

To evaluate the sensitivity of the qPCR assay using the designed primers, a plasmid vector (pUCFa) containing an artificially synthesized 900-bp fragment of the *A. butzleri* 16S rRNA gene was used as a standard. Ten-fold serial dilutions from 6.4×10^6^ to 6.4×10^0^ copies μL^−1^ were prepared, as well as standards containing 5.0×10^1^, 4.0×10^1^, 3.0×10^1^, 1.0×10^1^, 5.0×10^0^, 3.0×10^0^, and 1.0×10^0^ copies μL^−1^. qPCR was subsequently performed in a 25-μL reaction volume containing 2 μL of template DNA, 12.5 μL of a MightyAmp for Real Time (SYBR Plus, Takara Bio), 0.1 μL each of 50 pmol μL^−1^ of Arco-F and Arco-R primers, and 10.3 μL of ultrapure water. qPCR was run in a Thermal Cycler Dice Real Time System Single TP850 (Takara Bio). In each qPCR run, standard samples and negative controls were tested in triplicate under the following thermal conditions: 98°C for 2 min, followed by 45 cycles at 98°C for 10 s, 55°C for 30 s, and 68°C for 40 s. Following amplification, a melting curve analysis was performed to identify a specific single peak melting temperature (*T*_m_) using the following thermal conditions: at 95°C for 15 s, at 60°C for 30 s, and at 95°C for 15 s. Threshold cycle (*C*_T_) values were measured using the second derivative maximum method and TP850 software version 2.0 (Takara Bio).

### Application of the qPCR assay to quantify 16S rRNA genes of *Arcobacter* spp. in different water sources and bacterial 16S rRNA genes

In addition to the 15 samples analyzed for NGS, 33 samples were collected from shallow dug wells (*n*=15), deep tube wells (*n*=4), shallow tube wells (*n*=6), stone spouts (*n*=5), and springs (*n*=3) in the Kathmandu Valley. All procedures for sample collection and bacterial DNA extraction were the same as those described for the initial 15 water samples. The thermal conditions, qPCR mixture components, and qPCR thermal conditions used to quantify the 16S rRNA genes of *Arcobacter* spp. were the same as those described earlier for standard samples, except for the number of cycles used, which was 35.

The quantification of total bacterial 16S rRNA genes was performed by qPCR using U515F and U806R primers with the same thermal conditions, qPCR mixture components, and qPCR reaction conditions as those described previously (4, 17, 44).

### Statistical analysis

The Wilcoxon signed-rank test was used to compare the abundance ratios of *Arcobacter* spp. detected by NGS and qPCR. Statistical analyses were performed using Microsoft Excel 2013 (Microsoft, Redmond, WA, USA). Significance was set at *P*<0.05.

### Nucleotide sequence accession numbers

The 16S rRNA sequences obtained from the MiSeq gene sequencer and *Arcobacter* sequences from the clone library analysis were registered in the DNA Data Bank of Japan under the accession numbers DRA006433 and LC384241–LC384326, respectively.

## Results and Discussion

### Characterization of bacterial communities by NGS

The NGS analysis was used to examine bacterial community diversity in water samples. A total of 1,549,206 sequences were obtained from the 15 water samples analyzed, with a range of 91,283–115,833 sequences per sample. The bacterial community structure displayed 25 phyla, 63 classes, and 444 genera. Among the 63 classes identified, *Gammaproteobacteria* was identified as the most dominant in 9 water samples (KTM69, KTM73, KTM82, KTM86, KTM90, KTM99, KTM101, KTM105, and KTM106), with abundance ratios of 37.5–98.9%, whereas *Betaproteobacteria* was the most abundant in the remaining 6 samples, with abundance ratios of 36.3–52.5%.

Based on the criteria described in the Materials and Methods, 86 potential pathogenic bacterial genera were identified. Within these, 20 genera with an abundance ratio of >0.1% are shown in Table 1. Among the 15 samples tested, the genus *Acinetobacter* was the predominant potentially pathogenic genus in 13 samples, with abundance ratios of 7.47–78.95%. In the river water sample (KTM99) and shallow dug well sample (KTM94), *Arcobacter* and *Flavobacterium* were the predominant genera, with abundance ratios of 20.63 and 5.08%, respectively. Eight out of the 15 samples contained *Arcobacter* sequences with an abundance ratio of higher than 0.1%.

Among the 86 potential pathogenic bacteria, 20 genera exhibited abundance ratios of >0.1% in at least one of the 15 samples tested (Table 1). Of these 20 genera, the *Bacteroides*–*Prevotella* group is known to be associated with fecal contamination in environmental water (28), *Arcobacter* and *Sutterella* cause gastroenteritis through fecal-oral transmission (14), and pathogens such as *Flavobacterium* are associated with bacterial cold water disease and rainbow trout fry syndrome (43). *Acinetobacter*, *Clavibacter*, *Eubacterium*, *Flavobacterium*, *Pseudomonas*, and *Mycobacterium* are potentially opportunistic bacteria and are not associated with gastrointestinal infections through the ingestion of drinking water (51). Bacteria, such as *Chryseobacterium*, *Arthrobacter*, and *Comamonas*, are commonly found in soil and water (2, 8, 40), and *Sphingomonas* has been isolated from biofilms in drinking water systems (31). These potential pathogens, which may be naturally present in the environment, may be able to cause diseases in immunocompromised individuals following ingestion, contact, inhalation, and aspiration.

In the present study, *Acinetobacter* spp. were the most abundant potentially pathogenic bacteria in 13 out of the 15 samples tested, which agreed with the findings of our previous study conducted in the same region in a different year (17). Other studies also demonstrated the contamination of different pathogenic bacteria along with fecal indicator bacteria in groundwater samples in the Kathmandu Valley (17, 26, 38, 45, 48). Contamination by pathogenic bacteria may have adverse effects on human health and prompt action needs to be taken for the treatment of water samples containing these bacteria.

### Performance of the developed qPCR assay

Three bacterial DNA samples (KTM65, KTM99, and KTM106) were selected to test the specificity of the designed primers based on the abundance ratios of *Arcobacter* spp. obtained by the NGS analysis: the abundance ratios in KTM99 (20.63%) and KTM65 (6.54%) were the first and second highest values among the 15 tested samples, respectively, whereas *Arcobacter* spp. were not detected in KTM106 (Table 1). After performing conventional PCR and agarose gel electrophoresis, a band with a product length of approximately 600 bp was observed in the two samples designated as *Arcobacter* spp.-positive by NGS (KTM65 and KTM99), but not in the negative sample (KTM106).

Forty-three libraries were constructed using *E. coli* DH5α for each NGS-positive sample and a sequence analysis of all positive clones demonstrated that all the analyzed sequences exhibited a similarity of 94–100% to different species of *Arcobacter*, such as *A. aquimarinus*, *A. butzleri*, *A. cibarius*, *A. cloaecae*, *A. cryaerophilus*, *A. defluvii*, *A. ellisii*, *A. skirrowii*, and *A. suis*, as shown in Table 2.

To test the sensitivity of the new qPCR assay, a 16S rRNA gene DNA fragment of *A. butzleri* was used as the standard. As summarized in Table 3, the qPCR assay was able to amplify concentrations between 6.4×10^6^ to 1.0×10^1^ copies reaction^−1^ of the standard DNA in all triplicate samples, and even sometimes detected DNA down to 3 copies reaction^−1^. Mean *C*_T_ values were proportional to the log-transformed values of the standard DNA sample concentrations, with a correlation coefficient (*r*) of −0.998. The slopes of the standard curves ranged between −3.23 and −3.58, corresponding to amplification efficiencies of 91–104%. Amplification was not observed in negative controls. The lower quantification and detection limits were assessed to be 10 and 3 copies reaction^−1^, respectively.

The performance of the qPCR assay developed in the present study was judged by sensitivity and specificity testing. Previously designed primers have mostly focused on the detection of specific species of *Arcobacter* (*A. butzleri*, *A. cryaerophilus*, *A. cibarius*, *A. skirrowii*, *A. nitrofigilis*, *A. trophiarum*, *A. defluvii*, *A. ellisii*, *A. mytili*, and *A. molluscorum*) in different clinical samples, such as food, freshwater, seawater, sewage, sludge, and feces. In these cases, the detection of *Arcobacter* spp. was performed using various techniques, such as multiplex PCR (10, 12, 24), conventional PCR (5, 18, 19, 22), and real-time PCR (1, 6), and targeted different genes, such as 16S rRNA, *gyrA*, *rpo*B/C, and 23S rRNA.

Table 4 summarizes the comparison of our designed primers with primers previously designed by González *et al.* (18, 19), who targeted 16S rRNA genes of different species of *Arcobacter*. Out of 22 species of the genus *Arcobacter*, our designed forward and reverse primers completely matched with 19 and 21 of their 16S rRNA genes, respectively. Regarding the forward primer, Arco-F, one or two nucleotides mismatched to three species of *Arcobacter*, whereas only one nucleotide mismatched (to *A. nitrofigilis*) for the reverse primer, Arco-R. Although González *et al.* (19) designed forward and reverse primers using the sequences of only 10 species of *Arcobacter*, they completely matched to 19 and 17 species of *Arcobacter*, respectively. The primary aim of this study was to distinguish *Arcobacter* spp. from other closely related genera in the family *Campylobacteraceae*, and the designed primers have successfully targeted the wide range of *Arcobacter* spp. identified to date. In addition, the lengths of the amplified products produced when using the primers designed by González *et al.* were 85 (19) and 180 bp (18), whereas our primers amplified nearly 600 bp using MightyAmp for Real Time (SYBR Plus), a qPCR master mix specifically designed for the amplification of longer target sequences. This will enable us to perform the species identification of *Arcobacter* spp. using qPCR amplicons. In contrast, the qPCR assay developed in the present study was able to quantify down to 10 copies reaction^−1^ and sometimes detected levels as low as 3 copies reaction^−1^, which is equivalent to 200 copies mL^−1^ or 40 cells mL^−1^ of an original water sample. The qPCR assay described herein is expected to detect a broader range of *Arcobacter* spp. than previous assays (6, 18, 19, 32).

It is important to note that there was a single nucleotide mismatch found between the Arco-R reverse primer and *A. nitrofigilis* because this species was not considered in our initial assay design (Fig. 1). Therefore, a new reverse primer, Arco-R-rev (5′-GCAATCGGTATTCCTTCTGAT-3′), which has the same sequence as Arco-R, excluding the mismatched nucleotide at 3′, was designed and used in a qPCR reaction under the same conditions. The slope of the standard curves produced using Arco-F and Arco-R-rev was 3.58, indicating an amplification efficiency of 91% (data not shown). This result suggests that Arco-R-rev functions as efficiently as Arco-R and will be used in future surveys to quantify *Arcobacter* spp.

While previously designed primers are limited to the detection and quantification of only some species of *Arcobacter*, the primers developed here have proven to be broadly reactive and highly sensitive toward almost all known species of *Arcobacter*. This assay will be very beneficial in the future for the rapid and accurate quantification of *Arcobacter* spp. in environmental and clinical samples.

### Application of the qPCR assay to different water sources

**Fig. 2:**
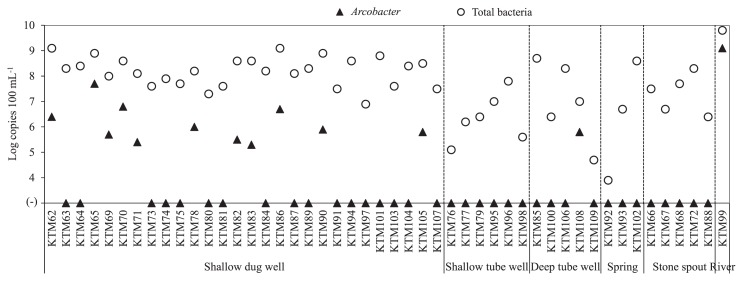
Concentrations of 16S rRNA genes of *Arcobacter* and total bacteria in water samples.

Fig. 2 shows the results of the quantification of 16S rRNA genes of *Arcobacter* spp. in 48 water samples using the new qPCR assay. The 16S rRNA genes of *Arcobacter* spp. were detected in 13 out of the 48 samples analyzed (27%) with concentrations higher than the limit of the quantification value (3.0 log copies 100 mL^−1^). Eleven out of the 28 shallow dug well samples tested (39%) were positive for the 16S rRNA genes of *Arcobacter* spp., with concentrations ranging between 5.3 and 7.7 log copies 100 mL^−1^. *Arcobacter* spp. were also detected in one out of the 5 deep tube wells tested and in the river water sample, with concentrations ranging between 5.8 and 9.1 log copies 100 mL^−1^, respectively. None of the samples from shallow tube wells, springs, or stone spouts were positive for *Arcobacter* spp.

After investigating its specificity and sensitivity, the qPCR assay was applied to additional water samples from different sources in the Kathmandu Valley. Thirteen out of the 48 water samples tested by qPCR (27%) tested positive for the 16S rRNA genes of *Arcobacter* spp. Using the concentration data for *Arcobacter* 16S rRNA genes and total bacteria, the abundance ratios of *Arcobacter* spp. by qPCR were estimated and compared to those obtained by NGS. *Arcobacter* spp. abundance ratios ranged between 0.18 and 19.2% in eight *Arcobacter* qPCR-positive samples, while ratios were below 0.004% in seven *Arcobacter* qPCR-negative samples. There was no significant difference in the abundance ratios of *Arcobacter* spp. obtained by NGS and qPCR (*P>*0.05; data not shown). Therefore, the abundance ratios obtained by the NGS analysis represent a reliable method for the estimation of *Arcobacter* concentrations in water samples.

In the Kathmandu Valley, 12% and 22% of groundwater are used for drinking and cooking purposes, respectively (41). The presence of *Arcobacter* spp. in groundwater samples may be directly associated with human health, namely, gastroenteritis, because they are transmitted by the fecal-oral route (46). *Arcobacter* spp. have not been tested in other water sources, such as piped, jar, and tanker water, which are more commonly used for drinking, cooking, and bathing purposes (41). Fecal indicator bacteria have been detected in these sources; therefore, contamination with pathogens capable of causing waterborne diseases is possible. *Arcobacter* spp. were detected at the highest concentration in the river water sample, which is widely used for several purposes, such as irrigation and washing vegetables prior to sale in the Kathmandu Valley. Activities that use polluted river water may pose diarrheal risks from waterborne pathogens, such as enteropathogenic *E. coli*, *Giardia*, and *Cryptosporidium*, via the consumption of raw contaminated vegetables (42). Waterborne outbreaks of gastroenteritis have also been linked to the contamination of *Arcobacter* spp. in well water, untreated drinking tap water, and the breakage of water distribution pipes (16, 27, 30).

The emerging pathogen, *A. butzleri*, is considered to be one of the potential enteric bacterial pathogens that are well-known to cause waterborne diseases, along with other bacteria, such as *Acinetobacter baumannii*, *Helicobacter pylori*, *Clostridium difficile*, *Listeria monocytogenes*, *Pseudomonas aeruginosa*, *Staphylococcus aureus*, and *Yersinia enterocolitica* (3). Different virulence genes in *Arcobacter* spp. are associated with adhesion, invasion, and cytotoxicity, which are pathogenic states of most of the species (33, 36). Since not all the species of *Arcobacter* may be human pathogens, the detection of these virulence genes in water samples may contribute to elucidating the role of *Arcobacter* in waterborne infections.

*Arcobacter* spp. have been recovered from environmental water samples, and the survival of *Arcobacter* has been observed at different temperatures and in non-chlorinated drinking water for up to 16 d, which also indicates the potential of *Arcobacter* spp. as pathogens (37, 39, 46). However, the inactivation of *Arcobacter* may be easily achieved by chlorination (39), which provides a simple way to eliminate them from various types of water sources, including drinking water.

## Conclusion

Eighty-six potential pathogenic bacterial genera were identified by NGS, and *Acinetobacter*, *Pseudomonas*, *Flavobacterium*, and *Arcobacter* were detected at a relatively high abundance. A highly specific and sensitive qPCR assay was developed to target most of the known species of the genus *Arcobacter*. The assay successfully detected *Arcobacter* spp. in different sources of water used for drinking and domestic purposes in the Kathmandu Valley. Thirteen out of the 48 samples analyzed (27%) were found to be positive for the 16S rRNA genes of *Arcobacter* spp. with concentrations ranging between 5.3 and 9.1 log copies 100 mL^−1^. Further studies are needed to detect the virulence genes of *Arcobacter* spp. in water samples, which may provide an actual assessment of the risks posed by these waterborne bacteria.

## Figures and Tables

**Table 1:** Distribution of potential pathogenic bacterial genera identified in water samples.

Potential pathogenic genus^a^	No. of authentic species	No. of BSL-2 or -3 species in ABSA^b^	Abundance in all sequences detected (%)^c^														

			Shallow dug well													Deep tube well	River

			KTM 64	KTM 65	KTM 69	KTM 73	KTM 82	KTM 83	KTM 84	KTM 86	KTM 89	KTM 90	KTM 94	KTM 101	KTM 105	KTM 106	KTM 99
*Acholeplasma*	18	7	—	—	—	0.06	0.02	0.01	<0.01	—	**0.12**	—	0.02	—	0.01	—	0.01
*Acinetobacter*	56	6	**28.75**	**15.70**	**35.24**	**28.55**	**33.09**	**11.88**	**7.47**	**18.39**	**31.01**	**38.41**	**0.96**	**25.74**	**78.95**	**61.65**	**10.25**
*Arcobacter*	22	2	0.01	**6.54**	**0.54**	0.06	0.02	0.04	**0.25**	**0.22**	**0.12**	0.06	**0.29**	0.01	**0.15**	—	**20.63**
*Arthrobacter*	91	3	<0.01	0.01	0.03	—	**0.11**	0.02	0.01	0.01	<0.01	0.01	—	<0.01	0.01	—	0.01
*Bacteroides*	96	36	<0.01	0.04	0.07	0.02	0.01	0.02	0.02	0.02	0.03	0.01	**0.27**	<0.01	**0.15**	—	**1.34**
*Chryseobacterium*	109	5	**0.41**	0.05	0.02	0.01	0.01	0.01	0.02	0.01	0.01	<0.01	0.02	0.01	<0.01	<0.01	0.06
*Clavibacter*	6	1	**0.13**	0.02	<0.01	**0.13**	0.08	<0.01	0.02	**0.44**	**0.13**	—	<0.01	0.01	—	—	0.06
*Comamonas*	22	1	0.06	**0.15**	0.04	0.10	0.08	**0.12**	0.09	0.03	0.02	0.02	0.01	0.06	0.01	—	**0.13**
*Dialister*	5	1	—	<0.01	—	—	—	—	<0.01	—	<0.01	—	0.07	—	0.05	<0.01	**0.62**
*Eubacterium*	53	23	—	—	—	—	<0.01	—	<0.01	—	<0.01	—	0.02	—	0.03	—	**0.11**
*Flavobacterium*	181	15	**2.30**	**11.47**	0.08	**3.79**	**0.39**	**3.99**	**0.66**	**4.35**	**6.91**	0.03	**5.08**	**0.65**	**0.11**	<0.01	**2.90**
*Sphingobacterium*	43	5	**0.19**	0.02	0.02	0.01	0.02	0.01	0.02	<0.01	0.01	<0.01	0.01	<0.01	0.01	—	0.01
*Megasphaera*	7	1	—	—	—	—	—	—	—	—	—	—	0.06	—	<0.01	—	**0.32**
*Mitsuokella*	3	1	—	—	—	—	—	—	—	—	—	—	0.01	—	<0.01	—	**0.27**
*Microvirgula*	2	1	<0.01	<0.01	**0.35**	<0.01	<0.01	0.01	0.01	<0.01	0.01	—	0.01	—	<0.01	—	0.06
*Mycobacterium*	188	43	<0.01	0.01	<0.01	0.02	0.07	0.02	0.06	0.02	0.02	**0.17**	**1.56**	<0.01	0.01	—	<0.01
*Prevotella*	50	18	—	0.01	<0.01	0.03	0.02	0.01	0.02	0.01	0.01	—	**0.58**	<0.01	**0.32**	—	**4.44**
*Pseudomonas*	240	11	**0.39**	**1.90**	**1.88**	**1.31**	**1.03**	**3.32**	**0.44**	**3.22**	**0.34**	**13.52**	**0.30**	**15.22**	**0.47**	0.03	**2.07**
*Sphingomonas*	111	2	0.06	0.06	0.05	0.02	**0.11**	0.02	0.03	0.01	0.02	0.01	0.05	0.01	0.01	—	<0.01
*Sutterella*	3	1	—	—	—	—	—	—	—	—	—	—	<0.01	—	<0.01	—	**0.11**

a Only potential pathogenic genera comprising >0.1% of sequences in at least one water sample are listed.

b BSL, bio-safety level; ABSA, American Biological Safety Association.

c Abundance ratio of >0.1% is indicated in bold type.

—, not detected. <0.01 indicates that at least one sequence was detected in the sample.

**Table 2:** Specificity of designed primers for *Arcobacter* spp. sequences.

Authentic species	Number of sequences identical to *Arcobacter* spp.		Similarity (%)

	KTM65	KTM99	
*Arcobacte aquimarinus*	12	4	98–99
*Arcobacter butzleri*	14	5	94–100
*Arcobacter cibarius*	0	1	99
*Arcobacter cloaecae*	15	2	99
*Arcobacter cryaerophilus*	0	24	99–100
*Arcobacter defluvii*	1	2	99
*Arcobacter ellisii*	1	0	99
*Arcobacter skirrowiii*	0	4	94–99
*Arcobacter suis*	0	1	99

Total	43	43	

**Table 3:** Concentrations of standard DNA samples of *Arcobacter* spp. and C_T_ values obtained from the qPCR assay.

Concentrations of standard samples (copies reaction^−1^)	No. of positive wells/no. of tested wells	C_T_ value (mean±SD)
6.4×10^6^	3/3	12.9±0.0
6.4×10^5^	3/3	16.2±0.4
6.4×10^4^	3/3	19.1±0.4
6.4×10^3^	3/3	23.1±0.4
6.4×10^2^	3/3	26.9±0.2
6.4×10^1^	3/3	30.4±0.4
5.0×10^1^	3/3	31.2±0.2
4.0×10^1^	3/3	31.6±0.5
3.0×10^1^	3/3	31.4±0.5
2.0×10^1^	3/3	33.0±1.0
1.0×10^1^	3/3	32.4±0.3
6.4×10^0^	2/3	33.9±1.0
5.0×10^0^	3/3	35.7±0.4
3.0×10^0^	2/3	33.2±0.0
1.0×10^0^	0/3	Not determined
0	0/3	Not determined

**Table 4:** Comparison of primers targeting 16S rRNA genes of the genus *Arcobacter*.

Species	No. of nucleotide mismatches					

	This study		González *et al.*, 2014 (19)		González *et al.*, 2000 (18)	
		
	Arco-F (22 nt)	Arco-R (22 nt)	Arco-Fw (21 nt)	Arco-Rv (26 nt)	Arc1 (22 nt)	Arc2 (22 nt)
*Arcobacter butzleri*	0	0	0	0	1	0
*Arcobacter cryaerophilus*	0	0	0	0	1	0
*Arcobacter thereius*	0	0	0	0	1	1
*Arcobacter trophiarum*	0	0	0	0	0	0
*Arcobacter anaerophilus*	0	0	2	0	3	6
*Arcobacter aquimarinus*	0	0	0	0	1	7
*Arcobacter bivalviorum*	0	0	2	0	6	7
*Arcobacter cibarius*	0	0	0	0	0	0
*Arcobacter cloacae*	0	0	0	0	1	7
*Arcobacter defluvii*	0	0	0	0	1	7
*Arcobacter ebronensis*	1	0	2	0	3	6
*Arcobacter ellisii*	0	0	0	0	1	7
*Arcobacter halophilus*	0	0	0	1	1	4
*Arcobacter lanthieri*	0	0	0	0	1	1
*Arcobacter marinus*	1	0	0	0	3	6
*Arcobacter molluscorum*	0	0	0	1	1	6
*Arcobacter mytili*	0	0	0	1	1	4
*Arcobacter nitrofigilis*	0	1	0	0	2	6
*Arcobacter pacificus*	2	0	0	0	3	6
*Arcobacter skirrowii*	0	0	0	0	1	0
*Arcobacter suis*	0	0	0	1	1	7
*Arcobacter venerupis*	0	0	0	1	1	6

No. of completely matched species	19	21	19	17	2	5

Amplified product size (bp)^a^	624		83		180	

a Sizes are based on the sequence of the *A. butzleri* 16S rRNA gene.
